# Boosting Mitochondrial Potential: An Imperative Therapeutic Intervention in Amyotrophic Lateral Sclerosis

**DOI:** 10.2174/1570159X20666220915092703

**Published:** 2023-04-12

**Authors:** Swati Dhasmana, Anupam Dhasmana, Sudhir Kotnala, Varsha Mangtani, Acharan S. Narula, Shafiul Haque, Meena Jaggi, Murali M. Yallapu, Subhash C. Chauhan

**Affiliations:** 1 Department of Immunology & Microbiology, School of Medicine, University of Texas Rio Grande Valley, McAllen, Texas, TX, USA;; 2 South Texas Center of Excellence in Cancer Research, School of Medicine, University of Texas Rio Grande Valley, McAllen, TX 78504, USA;; 3 Himalayan School of Biosciences, Swami Rama Himalayan University, Dehradun, India;; 4 Narula Research LLC, 107 Boulder Bluff, Chapel Hill, North Carolina, NC 27516, USA;; 5 Research and Scientific Studies Unit, College of Nursing and Allied Health Sciences, Jazan University, Jazan, 45142, Saudi Arabia;; 6 Centre of Medical and Bio-Allied Health Sciences Research, Ajman University, Ajman, United Arab Emirates

**Keywords:** ALS, mitochondrial dysfunction, neurodegeneration, ROS in ALS, excitotoxicity, mitochondrial biogenesis, mitochondrial reactivation

## Abstract

***Background*:** Amyotrophic Lateral Sclerosis (ALS) is a progressive and terminal neurodegenerative disorder. Mitochondrial dysfunction, imbalance of cellular bioenergetics, electron chain transportation and calcium homeostasis are deeply associated with the progression of this disease. Impaired mitochondrial functions are crucial in rapid neurodegeneration. The mitochondria of ALS patients are associated with deregulated Ca^2+^ homeostasis and elevated levels of reactive oxygen species (ROS), leading to oxidative stress. Overload of mitochondrial calcium and ROS production leads to glutamate-receptor mediated neurotoxicity. This implies mitochondria are an attractive therapeutic target.

***Objective*:** The aim of this review is to brief the latest developments in the understanding of mitochondrial pathogenesis in ALS and emphasize the restorative capacity of therapeutic candidates.

***Results*:** In ALS, mitochondrial dysfunction is a well-known phenomenon. Various therapies targeted towards mitochondrial dysfunction aim at decreasing ROS generation, increasing mitochondrial biogenesis, and inhibiting apoptotic pathways. Some of the therapies briefed in this review may be categorized as synthetic, natural compounds, genetic materials, and cellular therapies.

***Conclusion*:** The overarching goals of mitochondrial therapies in ALS are to benefit ALS patients by slowing down the disease progression and prolonging overall survival. Despite various therapeutic approaches, there are many hurdles in the development of a successful therapy due to the multifaceted nature of mitochondrial dysfunction and ALS progression. Intensive research is required to precisely elucidate the molecular pathways involved in the progression of mitochondrial dysfunctions that ultimately lead to ALS. Because of the multifactorial nature of ALS, a combination therapy approach may hold the key to cure and treat ALS in the future.

## 1 INTRODUCTION

Amyotrophic Lateral Sclerosis (ALS) is a fatal, progressive neurological disorder characterized by the death of motor neurons in the brain and spinal cord [1]. ALS is now gaining recognition as a multisystem neurodegenerative disorder because of its heterogeneity at clinical, genetic, and neuropathological levels [2]. It exists in two forms, familial (fALS) and sporadic (sALS). Only 5-10% of cases are fALS, and the remaining 90-95% are sALS with an unknown etiology [3]. About 50 genes have been identified, which are referred to as ALS-related genes. Among these SOD1, C9orf72, fused in sarcoma (FUS), and TDP-43 are the most common ALS-related genes [4]. Muscle weakening is a common attribute of ALS. In bulbar-onset disease, the weakening starts in facial, tongue and pharyngeal muscles, resulting in dysarthria (speech abnormality) followed by dysphagia (swallowing disorder). In spinal onset disease, muscle weakening starts in distal upper-limb or lower-limb muscles. The weakness spreads over time to contralateral, rostral and caudal sides from the point of origin [5]. Age on onset, site of onset and progression rate are highly variable in ALS individuals. However, it is ruthlessly progressive in most cases, with survival of approximately 3 years after the symptom onset, only a small fraction of patients survive for 10 years or more [6]. Death in ALS patients is mostly attributed to respiratory failure [7]. ALS has a global incidence of 1.59 and a prevalence of 4.42 per 100000 individuals. Standardized incidence of ALS in South and North America is 1.59 and 1.79 per 100000, respectively [8]. The age group with the highest risk of ALS development ranges between 45-75 years. Cumulative lifetime risk of developing ALS is 1:400 in women and 1:350 in men, which indicates that ALS prevalence is more among men than women [2].

## 2 UNDERLYING PATHOPHYSIOLOGY OF ALS

Loss of neuromuscular connection, axonal retraction, death of motor neurons, astrogliosis, microgliosis, and presence of ubiquitin positive inclusions are the neuropathological signatures of ALS [9]. ALS is a multifactorial disease, and several molecular pathways are implicated in its pathogenesis. Some common pathophysiology underlying ALS include protein aggregation, excitotoxicity, neuroinflammation, mitochondrial dysfunction and oxidative stress, oligodendrocyte dysfunction, cytoskeletal disturbances and axonal transport defects, disturbed RNA metabolism, nucleocytoplasmic transport deficits, and impaired DNA repair [10, 11] and depicted in Fig. (**1**) [12].

Protein aggregation and its impaired degradation contributes to ALS. Protein aggregation impairs normal protein homeostasis and generates cellular stress. The misfolded proteins overburden the cell and are subjected to ubiquititination/autophagy. In ALS, many genes involved in the protein degradation pathway are mutated, such as Ubiquilin-2 (UBQLN2), sequestosome 1 (SQSTM1), Optineurin (OPTN), TANK Binding Kinase 1 (TBK1), Valosin Containing Protein (VCP), and chromosome 9 open reading frame 72 (C9orf72) [2]. The dysregulation of autophagy leads to the protein accumulating in the cell. The misfolded and aggregated proteins damage the neighboring cells *via* extracellular vesicle mediated transfer; thus, ALS has prion like propagation [13, 14]. Similar to the proteins, there is an accumulation of neurofilaments (NFs) too. In the ALS condition, NFs undergo abnormal phosphorylation, which alters their axonal transport and results in their accumulation in cell bodies and proximal axons [15]. Oxidative stress holds the capability to cause dysfunction in all components of the cell. Oxidative stress occurs when antioxidant defenses are not at optimum levels or when there is the presence of increased generation of ROS [16]. ROS damages cell structures as well as RNA/DNA/proteins. Superoxide dismutase 1 (SOD^1^] protects the cell from oxidative damage, but in the ALS condition, SOD1 is mutated. This leads to the loss of function and gain of toxic function of SOD1 [17]. In ALS, SOD1 mutation is not only confined to motor neurons but extends to glial cells. This switches the anti-inflammatory/neuroprotective relationship of motor neurons and glial cells to pro-inflammatory/neurotoxic. Thus, leading to neuroinflammation [18, 19]. Glutamate, produced in the presynaptic terminal, is an excitatory neurotransmitter. It triggers an action potential. Excessive activation of glutamate receptors leads to glutamate excitotoxicity which results in the death of neurons [20]. Certain RNA metabolism genes are mutated in ALS, such as TAR DNA binding protein (TARDBP), fused in sarcoma (FUS), heterogeneous nuclear ribonucleoprotein A1 (HNRNPA1), HNRNPA2B1, and so on. Mutated TARDBP and FUS result in aberrant stress granule, pathogenic RNA foci, aberrant nucleocytoplasmic shuttling, *etc*. [21, 22]. Endoplasmic reticulum (ER) is the site for protein folding. During ALS, the normal function of the ER is disrupted. Both ER and mitochondria are involved in calcium homeostasis and lipid synthesis. Also, they have a physical and functional connection. Therefore, it is suggested there is a connection between ER stress and mitochondrial dysfunction in ALS as there is cross-talk between both organelles [23]. In the ALS condition, there are various changes observed in mitochondria, morphologically like dilation, distortion, vacuolation, *etc*., and physiologically such as abnormal ATP/ROS production, impaired energy homeostasis, calcium homeostasis, apoptosis, and axonal transport [24].

Based on the previous studies, it is evident that mitochondrial dysfunction plays a crucial role in ALS pathogenesis. This review focusses on the detailed discussion about mitochondrial dysfunction, its implication, process, genes associated, and mitochondrial medicine.

## 3 MITOCHONDRIAL DYSFUNCTION AS THE FOUNDATION OF ALS PATHOPHYSIOLOGY

Mitochondria are the powerhouse of the cell, their dysfunction results in insufficient energy production that cannot meet the energy requirement of the organ system, such as in the nervous system [25]. Mitochondria play an essential role in motor neuron (MN) survival. Morphological and functional changes are observed in various neurological diseases, including ALS [26-28]. Most evidence of mitochondrial dysfunction are based on the disease model. However, postmortem studies also reflect mitochondrial alterations in the medulla of ALS patients where mitochondria show the abnormal distribution in the soma or proximal axons of MNs [29]. This abnormal distribution may be attributed to altered mitochondrial trafficking. Newly generated mitochondria move from the cell body to distal segments of neurite and damaged mitochondria move towards the cell body for degradation. Kinesin and dynein motors mediate the anterograde and retrograde movement of mitochondria, respectively. Normally, the Miro1-Milton adaptor complex indirectly links Kinesin and dynein motors with mitochondria. In the spinal cord of ALS patients, significantly reduced expression of Miro1 is observed. Similar observations were made in the brains of transgenic mice with mutated SOD1/TDP43.

This indicates that Miro1 deficiency results in mitochondrial movement discrepancies in ALS and ALS experimental models [30]. Along with abnormal distribution, mitochondria exhibit characteristics such as swollen, vacuolated and with lesser DNA [31], the lesser activity of electron transport chain complexes, as well as mitochondrial enzymes [32]. The vacuoles in the mitochondria develop due to the expansion of mitochondrial intermembrane space, as well as the outer membrane. Peroxisomes are also involved in the vacuolation process. Peroxisomes play a pivotal role in the ether phospholipids and cholesterol synthesis. During vacuolation, the expansion of the mitochondrial outer membrane requires

a new membrane. Peroxisomes provide new membranes either by lipogenesis or fusing themselves with the vacuolar membrane [26]. Swelling of mitochondria results from the opening of the mitochondrial permeability transition pore (mtPTP), which allows the entry of water and solutes. Under normal conditions, the high membrane potential of mitochondria keeps the mtPTP closed, however in ALS condition, due to oxidative/nitrosative stress, the mitochondrial membrane potential drops, resulting in the opening of mtPTP [28]. The pictorial illustration of mitochondrial dysfunction is mentioned in Fig. (**2**).

### 3.1 Mitochondrial Damage in Context to Gene Mutation

Mitochondrial dysfunction is attributed to various factors such as genetics, aging, oxidative stress, calcium dysregulation, mitochondrial quality control disruption, mitophagy, mitochondrial epigenetic alterations *etc*.

More than 160 mutations have been identified in the SOD1 gene. SOD1 aggregates were observed in the spinal cord mitochondria of the SOD1^G93A^ mouse model. Along with SOD1 aggregates, vesicles were also observed between outer and inner mitochondrial membranes. This led to altered mitochondrial respiration, energy production and increased oxidative stress [26, 33, 34]. Mutant SOD1 deposits in the intermembrane space and matrix of mitochondria, causing cytotoxicity. Mutant SOD1 deposited in the outer membrane is correlated with mitochondria-dependent apoptosis [29]. TDP43 is a DNA-binding protein. Its mutations in ALS lead to aberrant mitochondrial aggregation, fragmentation, lower mitochondrial membrane potential, and vacuolization. TDP43 affects mitochondrial dynamics by dysregulating mRNAs encoding proteins that have an active role in mitochondrial physiology. Mutant TDP43 also interferes with the mitochondrial respiratory chain by inhibiting oxidative phosphorylation of mitochondrial respiratory chain complex 1 by binding to its subsequent mRNA [31]. FUS is a DNA/ RNA binding protein. Mutated FUS protein accumulates in the cytoplasm and leads to cytotoxicity and mitochondrial dysfunction [30, 32]. Increased ROS production causes aberrant localization of FUS to mitochondria where it interacts with chaperones (heat shock proteins), resulting in mitochondrial fragmentation and increased ROS generation. FUS accumulation in mitochondria causes mitochondrial membrane potential depolarization, defects in axonal transport and energy generation [35]. Furthermore, FUS causes malfunction of DNA ligase 3, thereby hindering mtDNA replication and repair processes which ultimately leads to DNA

breakage junction defects and mitochondrial dysfunction [36]. C9orf72 is a poly arginine-rich dipeptide repeat (poly-GR and poly-PR) that preferentially binds to mitochondrial ribosomal proteins such as ATP5A1, which leads to mitochondrial degradation *via* ubiquitination [37, 38]. Additionally, slight fragmentation, hyperpolarization, increased ATP, mtDNA, mt-mass, PGC1-α protein, ROS, and fission/fusion imbalance was observed in fibroblast of C9orf72-ALS patients [39]. The mitochondrial coiled-coil-helix-coiled-coil-helix domain-containing protein 10 (CHCHD10) resides between the inner and outer mitochondrial membrane (at the contact point) [40]. Aberrant CHCHD10 protein results in deformed/extended mitochondrial cristae and mitochondrial structure, which are evident as reported in studies conducted on SOD1 and FUS mouse models [39, 41]. Accumulation of mutant SOD1 and its associated genes (Miro, Mfn2, Parkin, PGC1, p62, and CHCHD10) cause mitochondrial dysfunction that subsequently leads to neurodegeneration in ALS [31]. OPTN and TANK-binding kinase 1 (TBK1) regulate mitochondrial phagocytosis. In ALS, mutated OPTN and TBK1 accumulate in mitochondria where the ubiquitin-associated (UBA) structural domain of OPTN binds to the ubiquitin chain, promoting mitochondrial phagocytosis after attaching to microtubule associated protein 1A/1B-light chain 3 (LC3). Mutated OPTN and TBK1 result in abnormal mitochondrial accumulation, which ultimately leads to neuronal death [42].

### 3.2 Ageing and ROS Generation

Aging motor neurons develop the aberrant mitochondrial function. mtDNA mutations alone are insufficient to cause neurodegeneration. Aging motor neurons of wild-type mice exhibit altered excitability as well as axon membrane function, which are resultant of the altered expression of voltage-gated sodium channels [43, 44]. A decrease in muscle mitochondrial function was observed in aging wild-type mice, which correlated with decreased oxygen consumption rate and ROS production [45]. Mitochondria produce ROS as well as superoxide radicals. When the ROS exceeds moderate levels, it causes damage to DNA, proteins, and lipids, and ultimately cell death [46]. During ALS, ROS-induced mitochondrial dysfunction further promotes ROS generation in a positive feedback loop provoking neuronal death. Oxidative stress in ALS is a resultant of many signaling pathways as various genes (SOD1, TDP43, and C9orf72) are involved in the upregulation of oxidative stress markers [47, 48].

### 3.3 Aberrant Calcium Levels

An elevated level of calcium is associated with mitochondrial dysfunction as it causes aggregation of mutant proteins, depolarization of mitochondrial membrane and ROS generation. Excess calcium leads to ER stress causing protein misfolding as both mitochondria and ER play a role in calcium homeostasis [49, 50]. Impaired calcium flow between mitochondria and ER is the driving force for calcium release in FUS and TDP43 models of ALS [51, 52]. The main culprit for calcium dysregulation may be the mutation in the sigma-1 receptor (SigR1), located on the contact surface of mitochondria and ER, which is chiefly responsible for organelle calcium transport. This was supported by a study conducted in SigR1 knockout mice, where high cytoplasmic calcium levels and prolonged calcium recovery were observed, thus indicating mitochondrial and ER stress [53-55].

### 3.4 Deregulated Mitochondrial Homeostasis

Mitochondrial homeostasis is maintained by the removal of aberrant mitochondria through protein/vesicle degradation and phagocytosis [56, 57]. Ubiquitination is mediated by PTEN-induced putative kinase 1 (PINK1) and Parkin kinase in the Mitochondrial quality control (MQC) pathway. Ubiquitinated mitochondria have autophagy receptors (OPTN and p62/sequestosome-1, both substrates of TBK1) that bind to LC3 and mediate translocation to lysosomes for degradation [58-60]. Mutation in TBK1 restricts autophagy of abnormal mitochondria [61]. In ALS, a decreased autophagic flux is observed, which indicates less autophagic ability and subsequent accumulation of damaged mitochondria, resulting in deregulated mitochondrial dynamics and ROS generation in abundance [62]. Similarly, in ALS, a mutation in genes p62 and VCP interferes with mitochondrial phagocytosis [63].

The transcription factor EB (TFEB) is also involved in the MQC. TFEB regulates mitochondrial processes such as mitophagy (TFEB overexpression favors mitophagy, *i.e*., removal of damaged mitochondria), biogenesis (TFEB promotes the formation of new mitochondria mediated by activation of Peroxisome proliferator-activated receptor gamma (PPARγ) coactivator-1alpha (PGC-1α)), ROS scavenging (ROS can be eliminated by TFEB mediated pathways), and dynamics (fission and fusion) [64].

### 3.5 Epigenetic Modifications

Various studies indicate a reduction in the electron transport chain’s catalytic rate in the human brain underlying neurological disorders resulting in low ATP. Epigenetic modifications in mitochondrial DNA are associated with ALS. Epigenetic modification in cytosolic DNA, such as methylation and histone acetylation, controls mitochondrial biogenesis. This suggests the role of epigenetic modifications in mitochondria in neuronal dysfunction. Further research is required to elucidate the regulatory mechanisms governing mitochondrial epigenetics and its role in ALS [31].

### 3.6 Role of Neutrophils in Mitochondrial Dysfunction

One of the attributes of mitochondrial dysfunction in ALS is the release of mtDNA in the circulation, which mediates an immune response. Mitochondrial localization of TDP43^Q331K^ led to the mtDNA release in the cytoplasm resulting in the upregulation of the cGAS/STING pathway and fostering an inflammatory response. Also, the aggregation of TDP43^G298S^ in cytosol led to impaired mitophagy [65]. Impaired mitophagy results in mitochondrial accumulation as well as oxidative stress build-up and release of mtDNA. Circulating mtDNA acts as a signal and interact with immune cells such as macrophages and neutrophils [66, 67]. mtDNA is engulfed by neutrophils through endocytosis leading to activation of endosomal Toll-like receptor 9 (TLR9). TLR9 further activates transcription factor family nuclear factor kB (NF-κB), which in turn activates tumor necrosis factor-ɑ (TNF-ɑ). TNF-ɑ is associated with apoptosis. Also NF-κB further upregulates TNF-ɑ expression [65]. TLR9 also activates NOD-like receptor, pyrin containing protein 3 (NLRP3) d. NLRP3 further activates caspase1 (CAS1), which upon activation, mediates production of inflammatory cytokines such as interleukins (IL) -1β and 18 [68-70]. Aberrant protein aggregation induces NLRP3 inflammasome in the neural cells. NLRP3 mediated production of IL-1β and 18 induces pyroptotic cell death. Activation of NLRP3 inflammasome results in inhibition of mitophagy which further promotes mitochondrial destabilization and destruction [71].

Neutrophils can also be activated by mast cells. Mast cells are activated in response to damage. Activated mast cells release signals that mediate neurogenic inflammation. At the site of inflammation, mast cells increase vascular permeability and recruit the neutrophils that cause damage to neuromuscular cellular components. In the SOD1^G93A^ rat models, abundant mast cells and neutrophils were found around motor axons in the extensor digitorum longus muscle, sciatic nerve, and ventral roots. This shows that the entire peripheral motor pathway is infiltrated with the immune cells [72].

### 3.7 Non-neuronal Cells and Mitochondrial Dysfunction

Astrocytes are abundantly present glial cells in the brain. The glial cells in the brain confer neuroprotection are involved in processes like the release of trophic factors, protection against oxidative stress and excitotoxicity, providing essential metabolites and removal of damaging agents & cell debris. However, pertaining to neurodegeneration, glial cells get activated and, in turn, trigger the hypersecretion of proinflammatory factors, thereby causing neurotoxicity through neuroinflammation. Excessive mitochondrial fragmentation and dysfunction are characteristics of ALS. Mitochondrial fragmentation is the consequence of aberrant activation of mitochondrial fission, induced by excessive dynamin-related protein1 (Drp1). This leads to glial activation in animal models of neurodegenerative disorders. BV2, a microglial cellular model of SOD1^G93A^, exhibited mitochondrial fragmentation and dysfunction, decreased ATP production, increased mitochondrial as well as cellular ROS and decreased oxidative phosphorylation [73]. SOD1 mutation in ALS induces mitochondrial stress and promotes dysfunction and death of astrocytes and motor neurons. Mitochondria, in addition to energy production, also serve as a Ca^2+^ reservoir and influence intracellular signaling, and mutated SOD1 causes enhanced mitochondrial permeability transition pore (mPTP) and Ca^2+^ transient in astrocytes processes. The increased Ca^2+^ signaling in astrocytes induces the secretion of neurotoxic molecules, thereby accelerating neurodegeneration underlying ALS [74]. ALS is characterized by the progressive loss of motor neurons. Damage to non-neuronal cells leads to toxicity to the motor neurons. Primary astrocytes from rodent model of mutant hSOD1, induced motor neuron death in co-culture [75]. Similarly, induced astrocytes from C9orf72 and sporadic ALS (sALS), exhibited distinct metabolic profiles, such as loss of metabolic flexibility as compared to controls. However, loss of metabolic flexibility was not observed in the induced fibroblasts from C9orf72 and sALS. Loss of metabolic flexibility concerning adenosine, fructose, glycogen metabolism, and disruption in membrane transport of energy substrate in mitochondria led to enhanced starvation induced toxicity in astrocytes derived from C9orf72 [76].

Microglial cells (brain-resident macrophages) present in CNS work as immune surveillance, under steady-state condition survey for any foreign entities, thus maintaining homeostasis [77]. Upon encountering any disturbances in brain homeostasis, microglial cells undergo morphological changes and release cytokines/chemokines to restore homeostasis. Under progressive neurodegeneration and disruption of the blood-brain-barrier, other myeloid cells infiltrate into the brain and work together with resident microglial cells to mitigate the damage. The importance of non-neuronal cells in motor neurons is supported by a study where motor neurons without mSOD1 developed features of ALS pathology when surrounded by mSOD1-expressing glia, whereas motor neurons expressing mSOD1 but surrounded by WT glia appeared healthy. This suggests that microglia are chiefly involved in non-cell-autonomous motor neuron degeneration [78].

In light of the importance of non-neuronal cells, perivascular fibroblasts are recently identified as a unique cell type of the nervous system. They are present in between astrocyte and mural cell basement membranes and are chiefly involved in composing and remodeling basement membrane extracellular matrix. A study revealed accumulated marker proteins (SPP1 and COL6A1) of perivascular fibroblasts in enlarged perivascular spaces of the pre-symptomatic patients of sALS. Also, the increased SPP1 levels in the plasma of ALS patients at disease diagnosis predicted shorter survival more strongly as compared to the established risk factor of bulbar onset or neurofilament level in cerebrospinal fluid, which suggests that altered perivascular fibroblast activity precedes ALS disease onset [79].

## 4 MITOCHONDRIAL ASSOCIATED MEMBRANE (MAM) DYSFUNCTION

Metabolites and signaling molecules shuffle between organelles to maintain cell homeostasis. Cells communicate *via* membrane contact sites (MCS) [80]. In MCS, protein complexes tightly bind the membranes of two organelles facilitating the rapid, direct, and mutual transfer of signal between the compartments [81]. ER is the largest membrane bout organelle and forms contact with mitochondria, Golgi bodies, peroxisomes, endosomes, lysosomes, and plasma membrane. The MCS of ER and mitochondria is known as mitochondria-associated membranes (MAM). Structural and functional abnormalities of MAM are associated with ALS [82].

Neurons depend largely on the energy produced by mitochondria, especially those located at sites far away from the cell body (dendrites and axons). Approximately 125 million ATP molecules are required to transfer one vesicle along a 1 metre long axon of a human motor neuron [83]. This suggests that mitochondria in dendrites and axons are very important for neuronal survival. Along with mitochondria, ER is also involved in neuronal processes, which indicates that MAM are present in axons and dendrites. MAM are associated with cellular functions such as autophagy, mitophagy, calcium homeostasis, and phospholipid synthesis [84]. TDP43, FUS and SOD1 are typical pathological components of ALS. TDP43 overexpression interferes with calcium homeostasis by disrupting the association of VAPB and PTPIP51 mediated by activation of GSK-3β [52]. Similarly, FUS also disrupts VAPB/PTPIP51 association through GSK-3β activation [51]. Mutation in VAPB is also reported in ALS. A study shows that VAPB^56S^ overexpression restricts the binding of Miro to microtubules, thus affecting the mitochondrial axonal anterograde transport [85]. SOD1 is chiefly involved in oxidative stress and energy metabolism [86]. Voltage-dependent anion channel (VDAC), a mitochondrial pore protein, is a molecular target of SOD1. VDAC plays an important role in numerous mitochondrial functions, such as metabolite exchange, Calcium homeostasis, Calcium transfer through MAM, the permeability of ATP/ADP and the apoptosis pathway [87-89]. Blocking of VDAC by mutant SOD1 makes motor neurons vulnerable to injury from adjacent astrocytes, microglia, and non-neuronal cells. Although motor neuron damage is a characteristic of ALS, astrocytes and microglial damage promotes the progression of the disease [78, 82]. Another mutated gene in ALS pathogenesis is SigR1. SigR1 is present in MAM and its loss affects various processes like, reduced ER-mitochondrial contacts, disrupted Calcium homeostasis, altered mitochondrial dynamics, and activated ER Stress [54]. In the SOD1 mice model, disruption of SigR1 exhibited an accelerated onset of ALS and destruction of MAM structure [90].

## 5 MORPHOLOGICAL CHANGES IN MITOCHONDRIA

**Fig. (3):**
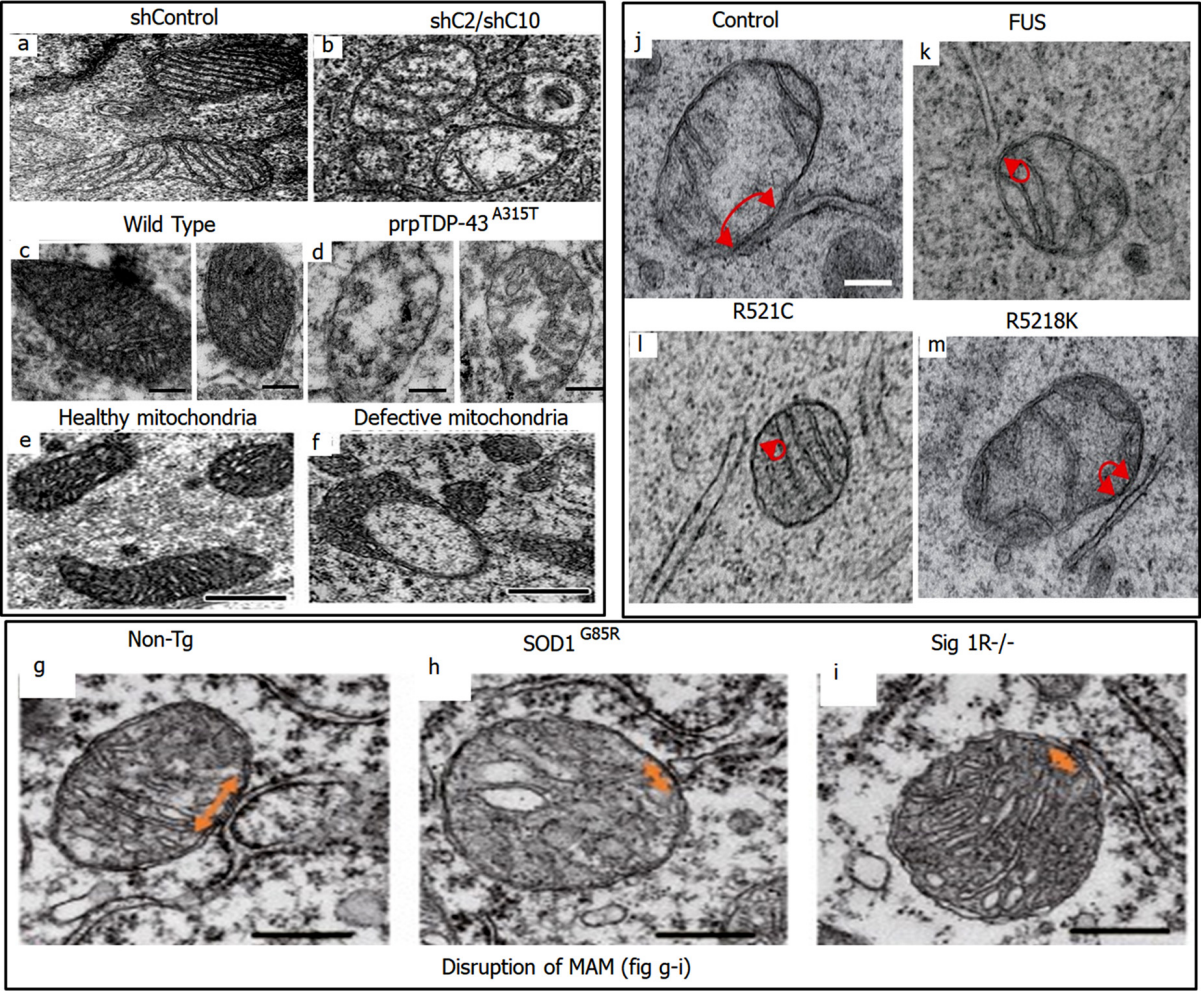
Morphological changes observed in mitochondria. (**a** and **b**) depict normal and abnormal cristae in mitochondria of control and CHCHD1/CHCHD10 silenced HeLa cells respectively. Reproduced from ‘Cell Death and Disease’, Volume 13, authored by Yu Ruan, *et al.* CHCHD2 and CHCHD10 regulate mitochondrial dynamics and integrated stress response. Copyright © The Author(s) 2022. Published by Nature; License Link: (https://creativecommons.org/licenses/by/4.0/). (**c** and **d**) depict the defects in mitochondria in TDP43 pathology within the corticospinal motor neurons (CSMN) of WT and prpTDP-43^A315T^ mice, respectively. Reproduced from ‘Scientific Reports’, Volume 12, authored by Mukesh Gautam, *et al.* [92] Mitochondrial dysregulation occurs early in ALS motor cortex with TDP-43 pathology and suggests maintaining NAD+ balance as a therapeutic strategy. Copyright © 2022, The Author(s). Published by Nature; License Link: (https://creativecommons.org/licenses/by/4.0/). (**e** and **f**) depict the healthy and defected mitochondria respectively in Corticospinal motor neurons (CSMN) at P15. Reproduced from ‘Cellular Neuropathy’, Volume 13, authored by Mukesh Gautam, *et al.* [93] Mitoautophagy: A Unique Self-Destructive Path Mitochondria of Upper Motor Neurons With TDP-43 Pathology Take, Very Early in ALS. Copyright © 2019 Gautam, Xie, Kocak and Ozdinler. Published by Frontiers in Cellular Neuroscience; License Link: (https://creativecommons.org/licenses/by/4.0/). (**g**-**i**) depict the MAM in WT, SOD1^G85R^ and SigR1 knockout mice model respectively. The arrows represent MAM area, and it is clearly observed that MAM area is reduced in SOD1^G85R^ and SigR1. Reproduced from ‘EMBO Molecular Medicine’, Volume 8, authored by Seiji Watanabe, *et al.* [90] Mitochondria-associated membrane collapse is a common pathomechanism inSIGMAR1- andSOD1-linked ALS. Copyright © 2016 The Authors. Published by EMBO Press; License Link: (https://creativecommons.org/licenses/by/4.0/). (**j**-**m**) also depict the MAM area in NSC34 cells transfected with CTRL, WT and FUS mutated gene vectors. The arrows represent the MAM area. Significantly reduced MAM area is observed in WT as well as mutant FUS expressed cells. Reproduced from ‘EMBO Reports’, Volume 17, authored by Radu Stoica, *et al.* [51] ALS/FTD-associated FUS activates GSK‐3β to disrupt the VAPB–PTPIP51 interaction and ER–mitochondria associations. Copyright © 2016 The Authors. Published by EMBO Press; License Link: (https://creativecommons.org/licenses/by/4.0/).

Mitochondria are subcellular organelles enclosed in a double membrane. Mitochondria undergo a continuous cycle of fission and fusion to maintain their shape, structure, and function. Mutation in CHCHD2 and CHCHD10 have been implicated in neurodegenerative disorders. These mutations result in defects of mitochondrial dynamics and cristae. Loss of CHCHD2 and CHCHD10 leads to abnormal cristae due to excessive processing of optic atrophy 1 (OPA1) (involved in the fusion process). CHCHD2 and CHCHD10 double knockdown HeLa cells exhibited cristae abnormalities when observed by transmission electron microscope (TEM) (Fig. **3a** and **b**) [91].

Neurons with TDP43 pathology, as well as upper motor neurons (UMNs) in ALS, exhibit mitochondrial defects at a cellular level. Similar mitochondrial defects are also observed in the corticospinal motor neurons (CSMN; a.k.a UMN in mice) of the TDP43 mouse model. In a study involving prp-TDP-43^A315T^ mice model, metabolic perturbations were investigated, and results showed reduced ATP synthesis and imbalance in the levels of NAD+, GSH (glutathione), and PEP (phosphoenol pyruvate). Fig. (**3c** and **d**) depicts the mitochondrial defects in the corticospinal motor neurons (CSMN) of prpTDP-43^A315T^ mice *vs* healthy wildtype (WT) [92]. Similarly, in a study aiming to investigate mitochondrial defects at an early stage, Corticospinal motor neurons (CSMN) from prpTDP-43^A315T^, hSOD1^G93A^, and PFN1^G118V^ mice at P15 (post-natal day 15) were subjected to approx coupled electron microscopy. The results indicated ultra-structurally defected mitochondria in CSMN of prpTDP-43^A315T^, hSOD1^G93A^, and PFN1^G118V^ mice as compared to mitochondria of non-CSMN Fig. (**3e** and **f**) [93].

MAM dysfunction is associated with ALS. SigR1 is a chaperone that is highly expressed in motor neurons and is present at the interface of ER and mitochondria. Loss of SigR1 reduces ER mitochondria cross talk and encourages neuronal degeneration in Sig1R-deficient mice, which signifies the importance of SigR1 in maintaining MAM integrity. Fig. (**3g**-**i**) depicts reduced ER-mitochondria contacted areas in motor neurons of 12-month-old SOD1^G85R^ and Sig1R^−/−^ mice as compared to non-transgenic mice, when observed by Electron micrographs [90]. Similarly, a study conducted by Stoica *et al.* shows significantly reduced ER and mitochondrial association in NSC34 cells transfected with FUS, FUS^R521C^ or FUS^R518K^ as compared to ctrl vector [51] Fig. (**3j**-**m**).

## 6 MITOCHONDRIAL FISSION AND FUSION DYNAMICS IN ALS

Aberrant mitochondrial morphology is well documented in ALS experimental models expressing ALS associated mutant SOD1 and TDP43. Mitochondria are dynamic as they constantly fuse and divide to either increase their energy supply (fusion) or to degrade mitochondria. This is known as mitochondrial dynamics, and it results in the change in mitochondrial member, morphology and size [94]. Mitochondrial dynamics is also important to maintain mitochondrial function. Under stress conditions, mitochondrial components are exchanged within the mitochondrial network to counteract damaged mitochondria (fusion) and the fission process helps create new mitochondria, thus maintaining the number of mitochondria. In ALS the balance between mitochondrial fusion and fission shifts towards fission. The fission and fusion processes are governed by numerous large dynamin related GTPases. The fission process involves dynamin-like protein 1 (DLP1or Drp1), Fis1, Mff, MiD49 & MiD51 and the fusion process involves Mitofusin1 (Mfn1), Mitofusin2 (Mfn2) & optic atrophy protein1 (OPA1). In the SOD1^G93A^ mice model, increased levels of fission and fusion regulators such as DLP1, Fis1, Mfn1 and OPA1 were observed before disease onset. Similarly, in the spinal cord of the TDP43 WT overexpression mice model, altered expression of fission and fusion regulators such as DLP1 and Mfn1 were observed [95]. This indicates the imbalance between mitochondrial fission and fusion processes in ALS.

## 7 GENDER BASED MITOCHONDRIAL DYSFUNCTION IN ALS

**Fig. (4):**
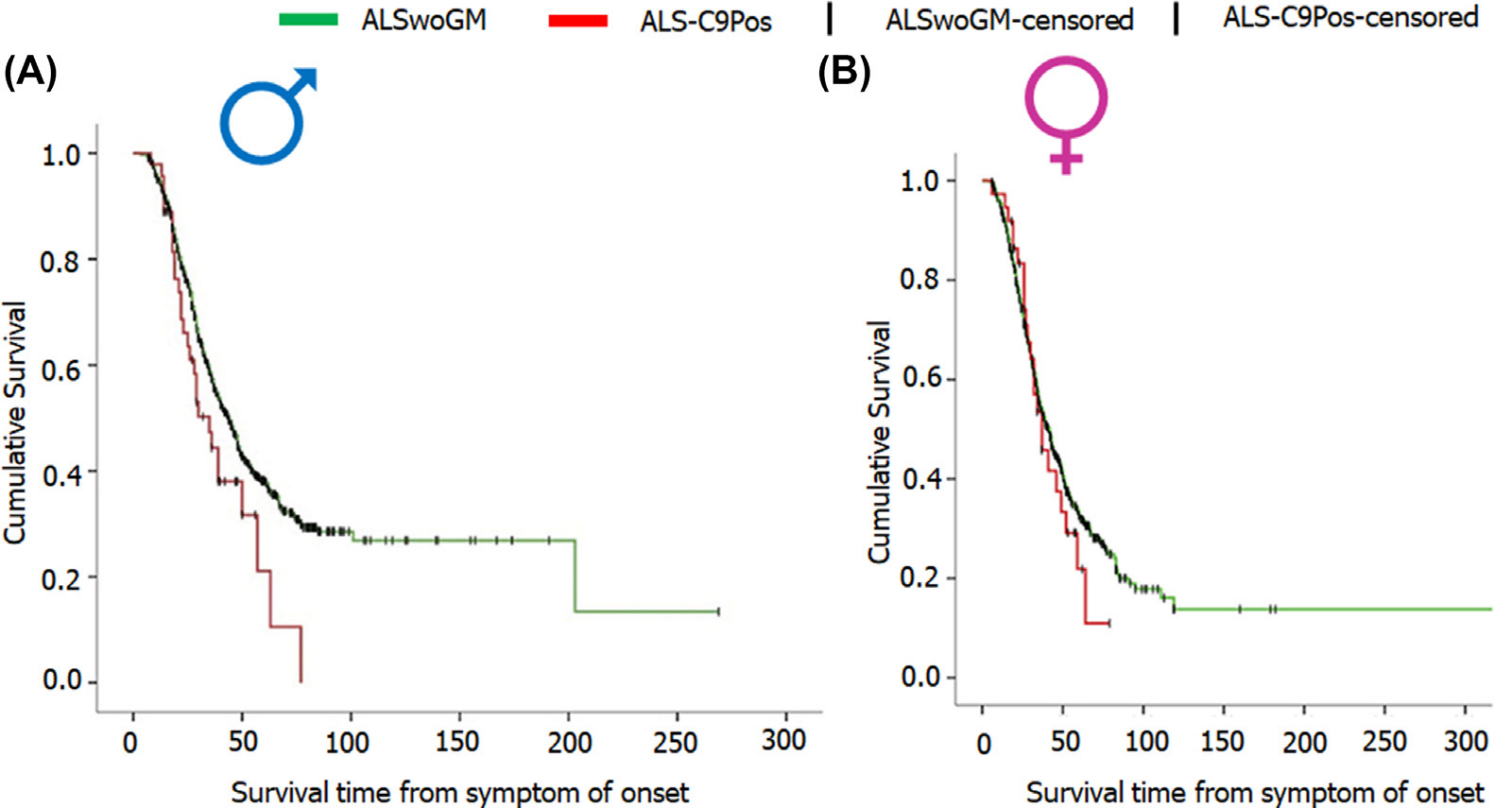
Representation of survival probabilities *via* Kaplan-Meier plots. (**A**) Graph A represents the male data showing median survival of 35 months for ALS-C9Pos (ALS patients carrying C9orf72 expansion) and 44 months for ALSwoGM (ALS patients without genetic mutation). (**B**) Graph B represents the female data showing median survival of 37 months for ALS-C9Pos and 42 months for ALSwoGM. Reproduced from ‘Frontiers in Neuroscience’, Volume 13, authored by Trojsi *et al*. [97] Comparative Analysis of C9orf72 and Sporadic Disease in a Large Multicenter ALS Population: The Effect of Male Sex on Survival of C9orf72 Positive Patients. Copyright © 2019. Published by Frontiers; open access article distributed under the terms and conditions of the Creative Commons Attribution (CC BY) license (https://creativecommons.org/licenses/by/4.0/).

ALS predominantly occurs in males than females [96], and show a shorter survival in males than females. According to a study conducted by Trojsi F *et al.*, Kaplan-Meier plots of survival probabilities show a median survival of 35 months in males with C9orf72 mutation and a median survival of 37 in females with C9orf72 mutation (graph shown in Figs. **4A** and **B**) [97], however, the difference is not significant.

The difference in ALS occurrence between males and females diminishes when the disease onsets at an older age which might be due to the declining oestrogen levels. Female ALS patients have been reported to have early menopause as compared to controls. Similarly, gender-based differences were also observed in SOD1^G93A^ female transgenic mice, which exhibited slower disease progression as compared to males. Ovariectomy resulted in accelerated progression of ALS and could be reversed with oestrogen treatment [98]. This implies that oestrogen has a protective role against ALS. SOD1 mutation underlying ALS leads to mitochondrial dysfunction in terms of calcium dysregulation. Oestrogen controls mitochondrial calcium handling *via* oestrogen receptors present in mitochondria. In females, oestrogen regulates calcium so that there is no calcium overload in the neural mitochondria. This mechanism is dependent on cyclophilin D (CypD). CypD regulates the calcium release through the permeability pore [99, 100]. In a study conducted by Daniel Cacabelos *et al.*, female SOD1^G93A^ mice showed better mitochondrial function as compared to their male counterparts when subjected to respirometric analysis (to analyze changes in respiratory chain function and ATP production) [101]. Misfolded SOD1 mislocalises to mitochondrial outer membrane as well as intermembrane space (IMS). Accumulation of misfolded proteins in IMS activates estrogen receptor alpha (Erα) axis of the mitochondrial unfolded protein response (UPR^mt^). Erα is predominantly found in females which suggest that the sexual dimorphism in ALS may be attributed to the sex hormones and their receptors [102]. Fig. (**5**) depicts the gender-based mechanism of mitochondrial dysfunction in neurological disorders [100].

## 8 ROLE OF NEUROHORMESIS IN NEURAL SYSTEM PROTECTION

Oxidative stress is a contributing factor in ALS pathogenesis. The increased oxidative stress leads to mitochondrial dysfunction. The brain cells have survival response networks that are regulated by redox-dependent genes (vitagenes), which include heat shock proteins (HSPs), sirtuins, thioredoxin, and lipoxinA4 (LXA4), Nrf2-dependent enzymes heme oxygenase and γ-glutamyl cysteine ligase. These genes detect cellular stresses such as redox perturbations and work towards cell survival under stress conditions [103]. At the initial stages of oxidative stress, neuroinflammatory process is modulated by inflammasome (multiprotein complex). Inflammasomes are formed in response to environmental stress, cellular damage, or infection. Inflammasome contains receptors for Pathogen/Damage Associated Molecular Patterns (PAMPs/DAMPs). Detection of DAMPs, PAMPs, AIM2 (absent in melanoma2) and/or NLRP3, lead to activation of inflammasome that result in pyroptotic and apoptotic cell death. AIM2 inflammasome is generally activated by cytosolic DNA. Mitochondria exhibit abundant DAMPs, thus triggering neuroinflammatory responses resulting in pyroptosis, apoptosis and autophagy. Inappropriate sensing of cytosolic DNA by AIM2 results in the development of numerous autoimmune/inflammatory diseases and neurodegenerative diseases [104, 105]. Such stresses can be pharmacologically modified using the phenomenon of hormesis. Hormesis refers to a dose-response trend characterized by low-dose stimulation and high-dose inhibition. Hormesis in the brain is a new emerging area of interest for the dose-response model. In the cellular models of neurodegenerative diseases, neurohormesis affects memory, learning, performance, antioxidants as well as oxidative stress-mediated neurodegenerative responses [106, 107]. Neuroprotective nutritional interventions involving antioxidants and anti-inflammatory agents can be promising against the oxidative stress-induced pathophysiology underlying neurodegenerative diseases [108]. The mode of action of polyphenol and nutritional mushrooms can be exploited under neurohormesis as both activate the heat shock protein (Hsp) pathway, which is a key player in the cellular stress response. Similarly, hemeoxygenase 1 (HO-1) has protective nature against oxidative and nitrosative stress, however, excessive HO-1 poses toxicity to cells. This implies that low doses of drugs, toxins or natural compounds can have a protective role, on the contrary, higher doses can promote toxicity [104, 109, 110].

## 9 MITOCHONDRIA AS THERAPEUTIC TARGETS IN ALS

Development of therapy against ALS is difficult because of its partially known etiology and ALS being a rare disease makes it difficult for large clinical trials. ALS has a multifaceted nature as various pathological pathways contribute to its pathophysiology. This complexity is the major hindrance in combating ALS. There are only 2 drugs (Riluzole and Edaravone) that have passed clinical trials and are being used to treat ALS despite they benefit a restricted population. Apart from these two drugs Albrioza is a new drug that has been approved by FDA in Canada for ALS and is under review in the USA. Lots of efforts are being made to relieve or prevent of ALS symptoms such as antisense oligonucleotides (ASOs), stem cells, CRISPR/Cas technique, non-invasive brain stimulation (NIBS) or ALS on a chip, synthesis, and screening of new compounds. However, all these efforts have not been fruitful, and ALS treatment has been restricted to palliative care. Therefore, it’s a need of the hour that new therapies should be developed to treat ALS [12].

Riluzole and Edaravone are the only FDA-approved drugs in the market for the treatment of ALS, which can extend the survival of patients up to a couple of months. The neuroprotective impact of Riluzole is because of its anti-glutamatergic effects. Still, this drug offers only a moderate advantage [111]. Riluzole is marketed as Rilutek by Sanofi. The mode of action of Riluzole in ALS is still unclear. Some evidence indicates that Riluzole has the capacity to diminish the ROS generation tendency through stimulation of glutathione synthesis [112]. Along with this effect, Riluzole was demonstrated to attenuate inward calcium current, which leads to protecting the axonal transport of neurofilaments. Mitigation of inward calcium currents is expected to release the pressure on cytosolic and mitochondrial calcium buffering mechanisms. This impact potentially reduces cytosolic calcium levels with knock-on effects on ROS generation and mitochondrial function [113]. More recently, Edaravone, also known as Radicava, a potent pyrazolone free radical scavenger and antioxidant, was also approved for the treatment of ALS. The precise molecular mechanism of Edaravone is still unknown, but the therapeutic potential of Edaravone may be due to its known antioxidant potential [114]. Edaravone’s neuro-protective effect may be observed by boosting prostacyclin production, reducing lipoxygenase metabolism of arachidonic acid by blocking hydroxyl radicals, preventing alloxan-induced lipid peroxidation and quenching active oxygen species [115]. One new FDA-approved oral drug in Canada is Albrioza. It is produced by Amylyx Pharmaceuticals. It is a combination of two drugs, sodium phenylbutyrate and taurursodiol, which, when combined, can safeguard neurons by preventing mitochondrial as well as endoplasmic reticulum dysfunction [116-118].

Mitochondria play a vital role in cells, and their dysfunction hampers various essential processes for neuronal survival. Due to the extensive predominance of mitochondrial dysfunctions, mitochondria are a hot target for various degenerative diseases like ALS. Prospective therapeutic approaches have been intended to reduce ROS generation, improve mitochondrial biogenesis and survival, inhibit apoptotic pathways, *etc*. [111]. On developing mitochondrial medicines for ALS, we need to be repurposing the available mitochondrial therapies for other indications. The available therapies, along with the specific classes, are mentioned in Table **1**.

These therapies (aiming at mitochondrial function/survival enhancement, and reduction in oxidative stress) have the potential against mitochondrial dysfunction in ALS. The animal trials using these approaches may have been successful, but the success could not be replicated in clinical trials. Animal trials with Coenzyme Q10, Dexpramipexole, Olesoxime and Creatine were successful but failed in human clinical trials. Similarly, Edaravone showed successful ROS scavenging effects in mouse models, but had a limited effect on humans. Also, minocycline, which is anti-apoptotic & anti-inflammatory, was successful in extending the survival of mouse models but was unsuccessful in the human phase III randomized trial. These episodes indicate the discrepancies between animal and human trials. These discrepancies may be attributed to the reasons such as lack of understanding of the target and its biology, lack of availability of relevant biomarkers indicating the efficacy/potency of the therapeutic interventions, dosage optimization, insufficient sample sizes, heterogeneous nature of ALS and absence of relevant disease model [145, 146]. Therefore, it is a requisite to work towards better therapy development. Like ALS, mitochondrial dysfunction is also multifactorial, therefore, it would be unlikely that a single therapy would be sufficient against all facets of dysfunction [43]. Mitochondrial disorders are not only confined to ALS but to a wide range of diseases such as Alpers syndrome, Autosomal dominant optic atrophy (ADOA), Coenzyme Q10 deficiency, Kearns-Sayre Syndrome (KSS), Leber hereditary optic neuropathy (LHON), Leigh syndrome, Mitochondrial encephalopathy with lactic acidosis and stroke-like episodes (MELAS), Myoclonic epilepsy with ragged redfiber (MERRF), Mitochondrial Neurogastrointestinal Encephalomyopathy (MNGIE), Neuropathy, Ataxia, Retinitis Pigmentosa (NARP), Non syndromic hearing loss (NSHL), Progressive external ophthalmoplegia (PEO) *etc*. [147]. These diseases might have overlapped pathophysiology with ALS with respect to mitochondrial dysfunction. Therefore, the use of mitochondrial medicines for these diseases may be extended to ALS depending upon the symptoms or pathophysiology. Some of the small molecules used in indications other than ALS are tabulated in Table **2** [148-165].

Despite the availability of these mitochondrial medicines/therapies, mitochondrial disease patients depend largely on exercise and dietary supplements. A large number of diseases fall under the category of mitochondrial disease, but very few respond positively to therapy. The reason behind not finding a successful treatment regimen is the disease being rare, clinically diverse, pathogenically complex, etiologically heterogeneous, and inadequate clinical trials [119]. Most of the treatments are palliative and symptomatic. Mitochondrial DNA (mtDNA) mutations also lead to mitochondrial disorders. mtDNA has a maternal inheritance. Therefore, the mutations in the mtDNA may be carried forward with generations. The mutated mtDNA may be homoplasmic (2 copies of mutated mtDNA; exhibiting severe symptoms) or heteroplasmic (one mutated, one wild-type copy of mtDNA; exhibiting non-existent to moderate symptoms). An asymptomatic mother may pass a vast amount of mutated mtDNA to the offspring due to the phenomenon of genetic bottlenecking of mtDNA. As the transmission of mutated mtDNA is unpredictable, preconception advice is impossible. Extensive research focuses on the prevention of the transmission of mutated mtDNA. However, ongoing research is progressing towards the prevention of mitochondrial diseases, but the development of new emerging therapies is required [166]. New approaches aiming at removing the mutated mtDNA are mitochondrial replacement therapy (MRT), gene therapy, correction of DNA heteroplasmy *etc*. Some of such therapies are tabulated in Table **3**. These therapeutic interventions have been proposed for indications other than ALS. However, they provide a scope to be used in ALS.

## CONCLUSION AND FUTURE DIRECTIONS

Mitochondria are the powerhouse of the cell and have a very important role in the survival of neurons. Mitochondrial dysfunction is observed on morphological as well as functional levels. Mitochondria related diseases are currently assumed to be the second most frequently detected inherited disease globally, and, unfortunately, there are even no established therapeutic portfolios. In ALS, mitochondrial dysfunction primarily includes enhanced ROS production, change in mitochondrial permeability and potential, decreased ATP generation, leaching of proapoptotic factors, *etc*. All these factors combined lead to synaptic dysfunction and, ultimately, neuronal death. A mitochondrion is a highly significant therapeutic target for ALS and many therapies have been implicated against mitochondrial dysfunction, as discussed in this review. However, there are many hurdles in the development of a successful therapy because of the multifaceted nature of mitochondrial dysfunction. Mitochondrial dysfunction is not the only pathophysiology underlying ALS, and it is difficult to say whether it leads to ALS or other pathophysiologies lead to mitochondrial dysfunction that ultimately leads to the development of ALS. This suggests that there is a need to fill the gaps, more intensive research is required that would shed light on the molecular pathways in the progression of mitochondrial dysfunction, and, ultimately ALS. The new therapeutic strategies should aim at activating/deactivating the metabolic pathways that would restore the normal functioning of the cells/neurons. The use of a single drug may not be sufficient in combating mitochondrial dysfunction and ALS because of their multifactorial nature. Therefore, the time of the single drug approach is gone, and now the time has come for a combination therapy approach which may hold the key to the future of curing ALS.

ALS is a disease where patients generally deal with defects in cellular energy metabolism (mitochondrial disease), which may lead to weak and inactive muscles. The weakness of muscle is directly linked to mitochondrial dysfunction, so there is an urgent need to work on therapies that can reactivate mitochondrial biogenesis. There are several therapeutic approaches which we have discussed in this review, but at the end of the story, biosimilar medicine can hold an important position in the reactivation of muscle activities. By keeping an eye on the chronic diseased status of ALS, we should focus on biological therapies. Chemical-based therapies can have an adverse impact on the vital organs (Liver and Kidney) of the body. While biological therapies can be used for a long time without hampering and compromising the normal functioning of vital organs. Along with these, gene or molecular medicines can be developed as personalized medicine for ALS patients where a physician can plan the therapeutic protocol according to the genetic variation of patients. They can use the MRT, Stem cell replacement, DNA correction, monoclonal antibodies against mutated proteins like SOD1 and supplementation of corrected fusion proteins. The careful biosimilar therapies selection is important because these kinds of therapies can be costly and somehow hazardous. The long‐term safety issues are still unknown, and ongoing monitoring is an important part of patient care.

## Figures and Tables

**Fig. (1):**
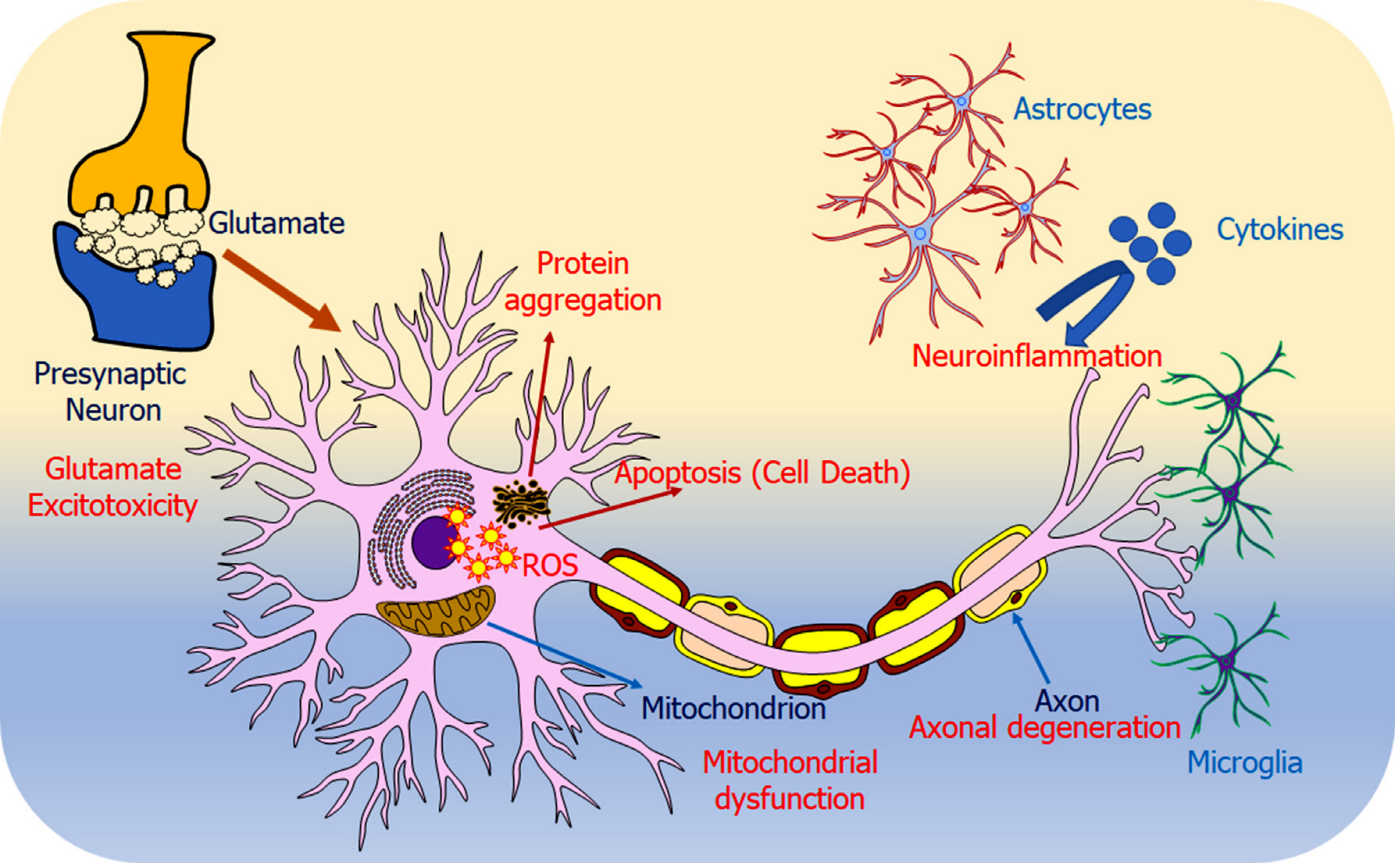
Graphical representation of overall pathophysiologies underlying ALS. The figure shows various disease mechanisms that are involved in motor neuron degeneration. Protein aggregation: Impaired proteostasis leads to aberrant protein aggregation, which may be due to the overload of the proteasome system and reduced autophagy. Glutamate excitotoxicity: Mutant EAAT2 (glutamate transporter) leads to accumulation of glutamate, which in turn hyperstimulates glutamate receptors, ultimately leading to extreme calcium influx. Neuroinflammation: Activation of astrocytes and microglia lead to more toxic molecule secretion as compared to neuroprotective molecules. Apoptosis: Motor neurons undergo apoptosis, thus, leading to neurodegeneration. Mitochondrial dysfunction: Mutated proteins mislocalize to mitochondria, thus, interfering with its normal functioning. Axonal degeneration: Changes in cytoskeletal organization, axonal transport and axonal outgrowth led to axonal degeneration, which result in axonal retraction from neuromuscular junction. Reproduced from ‘International Journal of Molecular Sciences’, Volume 23, authored by Sever B *et al*. [12] Comprehensive Research on Past and Future Therapeutic Strategies Devoted to Treatment of Amyotrophic Lateral Sclerosis. Copyright © 2022 by the authors. Published by MDPI.; open access article distributed under the terms and conditions of the Creative Commons Attribution (CC BY) license (https://creativecommons.org/licenses/by/4.0/).

**Fig. (2):**
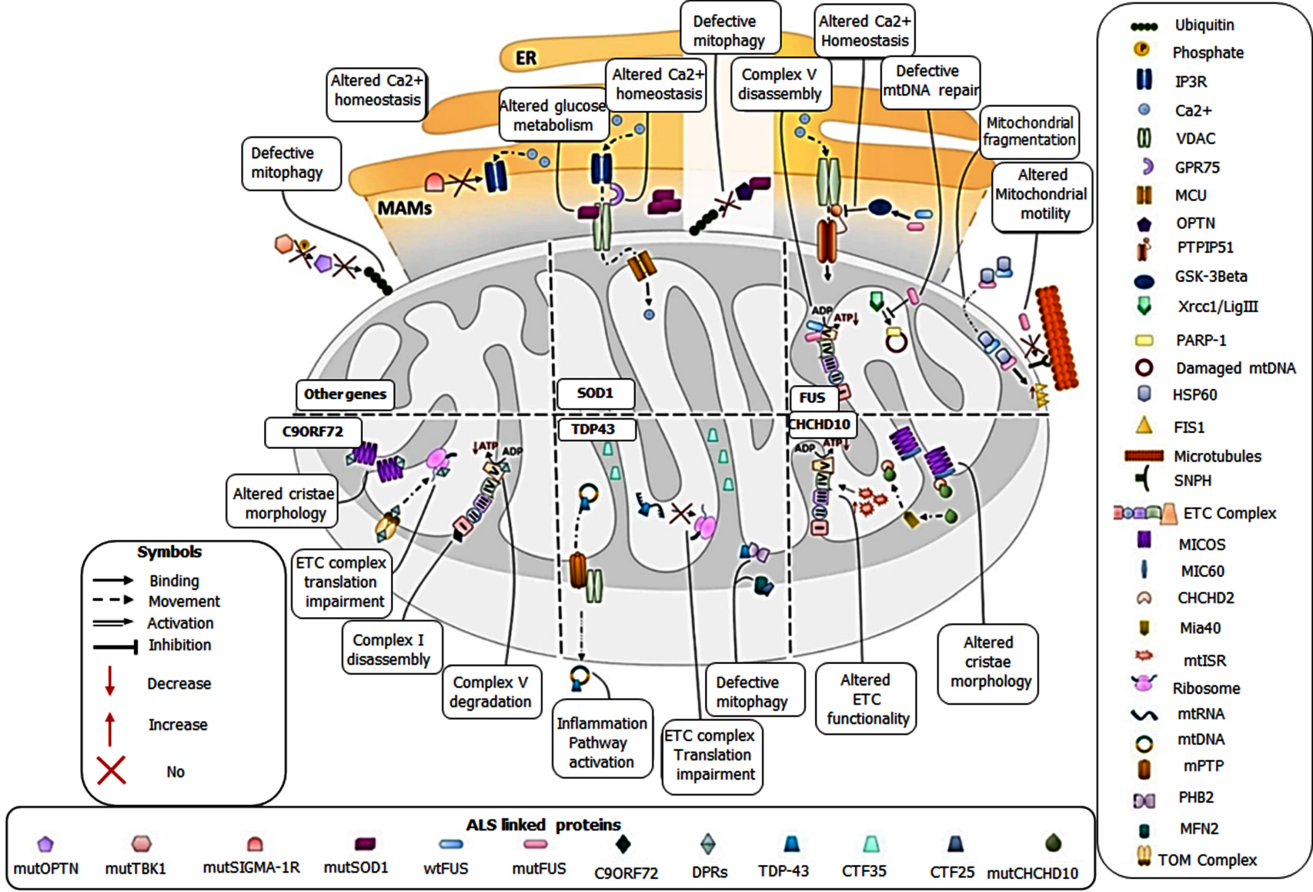
Graphical representation of consequences of mitochondrial dysfunction in ALS. Figure shows culprit genes of ALS and their associated pathways causing mitochondrial dysfunction. Other genes (tbk1, optn, sigma-1R) affect calcium homeostasis and mitophagy. SOD1 affects glucose metabolism, calcium homeostasis and mitophagy. FUS affects complex V assembly, mtDNA repair, mitochondrial motility/fragmentation, and calcium homeostasis. CHCHD10 affects cristae morphology and ETC function. TDP43 affects mitophagy, ETC complex translation and inflammation pathway. C9orf72 affects cristae morphology, ETC complex translation, complex I assembly and complex V degradation. Reproduced from ‘Metabolites’, Volume 12, authored by Niccolo Candelise, *et al*. Mechanistic Insights of Mitochondrial Dysfunction in Amyotrophic Lateral Sclerosis: An Update on a Lasting Relationship. Copyright © 2022 by the authors. Published by MDPI.; open access article distributed under the terms and conditions of the Creative Commons Attribution (CC BY) license (https://creativecommons.org/licenses/by/4.0/).

**Fig. (5):**
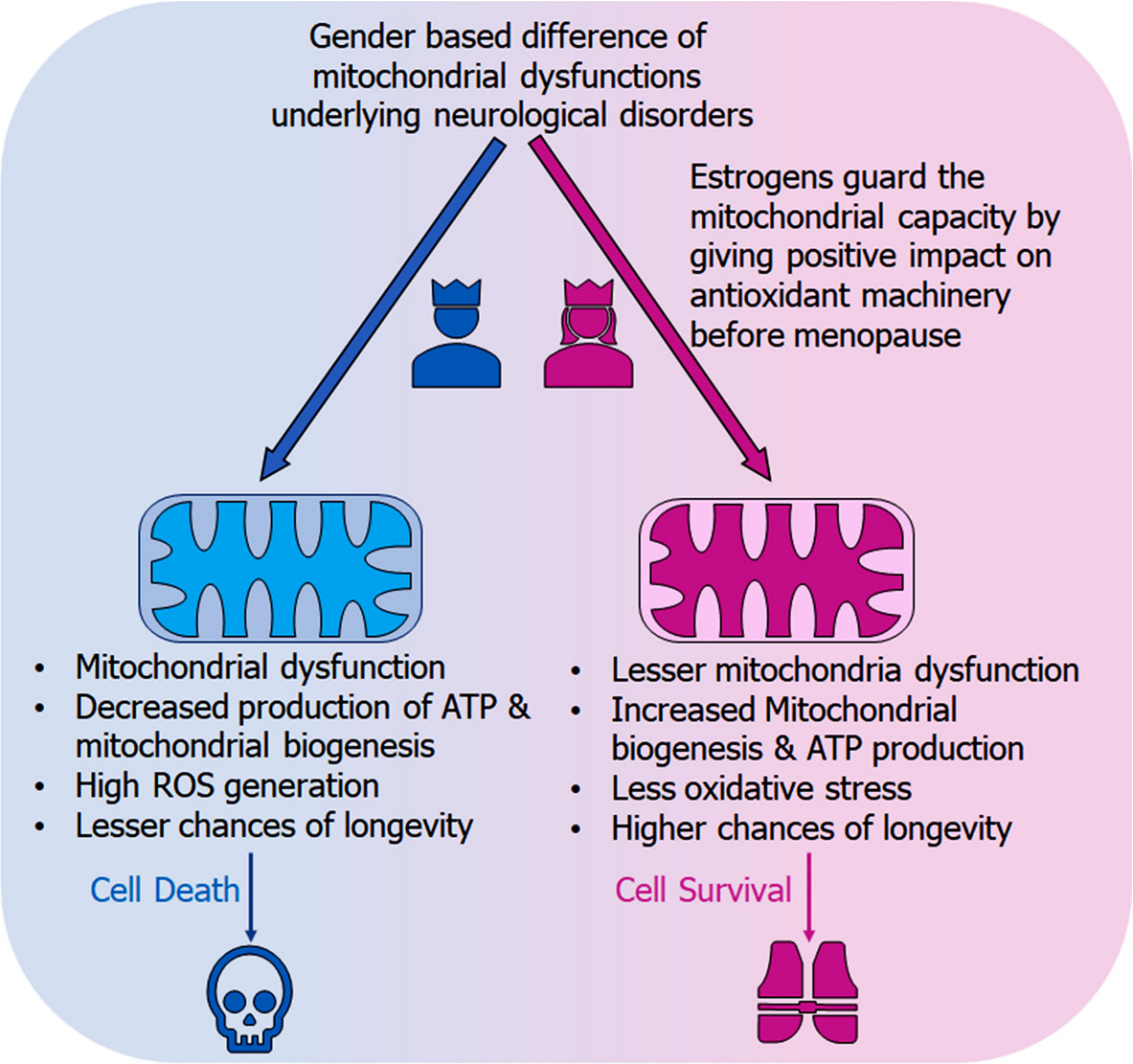
Graphical illustration displaying the gender-based discrepancies in the mitochondrial dysfunction w.r.t neurological disorders. The figure shows the protective role of oestrogen in females against mitochondrial dysfunction, whereas males, devoid of oestrogen are more prone to mitochondrial dysfunction and related pathophysiologies, including low ATP production, lesser mitochondrial biogenesis, thus overall shorter survival.

**Table 1:** Mitochondria based therapeutic interventions of ALS along with their molecular mechanism and disease conditions.

**S. No.**	**Interventions**	**Structure**	**Mechanism**	**Disease Condition**	**References**
1	Exercise or Aerobic endurance	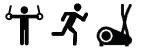	Mitochondrial biogenesis and increased muscle mitochondrial enzyme activities and muscle strength	ALS: Mitochondrial Diseases like Mitochondrial myopathy in ALS and other indications	[119-123]
2	Riluzole	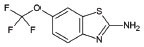	Diminishes the ROS generation *via* anti-glutamatergic effects tendency and attenuates inward calcium current	ALS: Maintaining mitochondrial calcium buffering mechanisms in ALS	[112, 113]
3	Edaravone	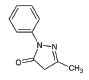	Neuro-protective effect by boosting prostacyclin production, reducing lipoxygenase metabolism of arachidonic acid by blocking hydroxyl radicals, preventing alloxan-induced lipid peroxidation and quenching active oxygen species	ALS: Free radical scavenger and antioxidant in ALS	[115]
4	Fenofibrate	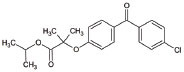	PPAR-α activation	ALS: Neuroinflammation and mitochondrial dysfunction in ALS	[124]
5	Vitamins C (Ascorbic Acid)	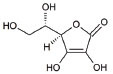	Endogenous defensive strategy against oxygen free radicals in mitochondria Protection against lipoperoxidation	ALS: Electron carrier and Antioxidant in ALS	[125]
6	Vitamin E (α-tocopherol)	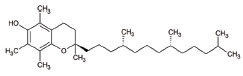	Protection against lipoperoxidation	ALS: Lipophilic antioxidant has the ability to cross cell membrane resulting in delay and lower risk of clinical onset in ALS Decrease in plasma levels of thiobarbituric acid reactive species	[126, 127]
7	Co-enzyme Q10 (CoQ10)	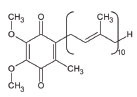	A strong lipid oxidants scavenger, which participates in transferring electrons from mitochondrial complexes I and II to mitochondrial complex III. CoQ10: stabilizes mitochondrial membrane potential, prevents cytochrome c release; inhibits mitochondrial permeability transition pore, and blocks. Bax translocation to mitochondria	ALS: Stabilized the motor neuron function in neuro-degenerative disorders like ALS and other indications	[128-131]
8	Glutathione (GSH)	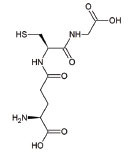	Antioxidant property: Neuronal GSH helps in the protection of neurons in the brain against ROS and oxidative damage	ALS: Regulates the development and progression of Amyotrophic Lateral Sclerosis	[132]
9	Resveratrol	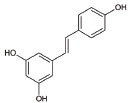	Inhibits mutant SOD1 (G93A) protein, up-regulates SIRT1, down-regulates AMPK/SIRT1 signalling and activates mitochondrial biogenesis	ALS: Improves muscle atrophy and mitochondrial dysfunction in ALS and other indications	[133-135]
10	β-carotene	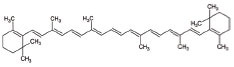	Antioxidant properties	ALS: Neuroinflammation and apoptosis in ALS.	[136]
11	7,8-Dihydroxy-flavone (7,8-DHF)	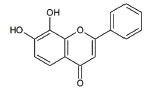	Modulates metabolic pathways and Improves motor deficits and enhances lower neuronal survival	ALS: Chronic administration of 7,8-DHF improved motor deficits in ALS	[137]
12	Epigallocatechin Gallate	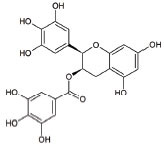	Protection against Lipoperoxidation, diminishes the level of NF-kB, caspase-3 and iNOS	ALS: Delays the advancement of ALS by alterations in intracellular signals, enhances survival signals (like PI3-K and Akt) and lowers death signals (like GKS-3ß, cytosolic cytochrome c, activated caspase3 and cleaved poly ADP-ribose polymerase) in ALS and other indications	[138-140]
13	Curcumin	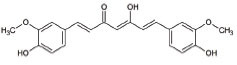	Activates Nrf2, reduces intra-cellular ROS levels, and eradicates excitability induced by TDP-43	ALS: Decreases ALS progression by reducing oxidative damage and improves survival, especially in bulbar onset patients	[140, 141]
14	Melatonin	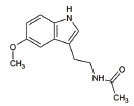	Antioxidant effect regulates mitochondrial dysfunction and bioenergetic function	ALS: Orally high doses delayed ALS progression *via* caspase-1/cytochrome c/caspase-3 cell death pathway, inhibits MT1 receptor loss and increases the survival rate	[141-143]
15	SBT-272	NA	Novel peptidomimetic, a role in mitochondrial energetics, by increasing ATP production and decreasing levels of reactive oxygen species (ROS) in dysfunctional mitochondria	Amyotrophic lateral sclerosis (ALS)	Stealth BioTherapeutics
16	MitoQ	-	Accumulates within mitochondria and protects it from oxidative damage	ALS: slowed the mitochondrial functional decline in the spinal cord and muscle; decreased nitroxidative damage in the nervous system; increased survival and slowed the progression of ALS symptoms.	[144]
17	Albrioza	-	Reduces neuronal death by blocking key cellular death pathways of mitochondria and endoplasmic reticulum	ALS: neuronal protection	[116-118]

**Table 2:** Mitochondria targeted agents (small molecules) in other than ALS indications.

**S. No.**	**Interventions**	**Structure**	**Mechanism**	**Disease Condition**	**References**
1	All-trans retinoic acid (ARTA/Retinoic acid)	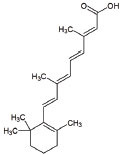	Retinoic acid used for Mitochondria biogenesis and stimulated the retinoid X receptor-α (RXRα) to repair the mitochondrial respiratory chain defect	Lipid metabolism	[119, 148]
2	KH176	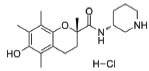	Intracellular reduction-oxidation-modulating compound used for the management of mitochondrial diseases	MELAS, LHON, Leigh, and other MDs	NCT02544217 & [149]
3	Bezafibrate	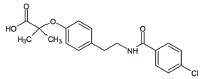	Mitochondrial biogenesis and PPAR-α activation	Mitochondrial myopathy	[150]
4	L-Arginine	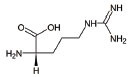	Improvement in aerobic capacity and muscle metabolism	Mitochondrial Encephalomyopathy, Lactic Acidosis, and Stroke-like syndrome (MELAS)	NCT01603446 & [151]
5	Rapamycin	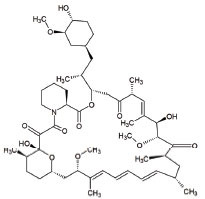	Improves motor endurance, muscle morphology, activation of autophagy *via* mTOR-dependent pathways and mitochondrial structure	Mitochondrial myopathy	[119, 152]
6	Vatiquinone or EPI-743	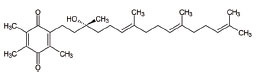	EPI-743 works by enhancing the regulation of cellular energy metabolism by directing NADPH quinone oxidoreductase 1 (NQO1)	Leigh Syndrome and congenital respiratory chain diseases	[153]
7	Idebenone	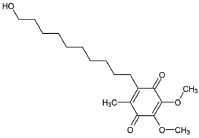	Idebenone stimulated the mitochondrial permeability transition pore (mPTP)	Leber Hereditary Optic Neuropathy (LHON)	[154]
8	Cysteamine bitartrate	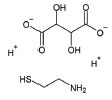	Antioxidant properties by enhancing glutathione biosynthesis	Mitochondrial respiratory chain disease	[155]
9	Omaveloxolone	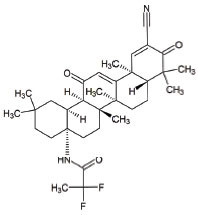	Omaveloxolone is an Nrf2-inducer, reduces Inflammation, provides protective shelter to mitochondrial depolarization, promoting mitochondrial respiration and preventing cell death	Friedreich’s Ataxia (FRDA): an autosomal recessive neurodegenerative disorder	[156]
10	Taurine	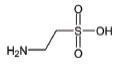	Neuroprotector by decreasing ER stress and antagonizing neurotransmitter receptors of GABAA, glycine and NMDA	Myopathy, encephalopathy, lactic acidosis, and stroke-like episodes (MELAS)	[157, 158]
11	L-Carnitine	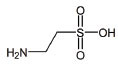	L-carnitine translocate the fatty acids into the mitochondrial compartment for β-oxidation. Role in carbohydrate metabolism like ketogenesis and glucogenesis. Elimination of toxic metabolites	Mitochondrial dysfunction, Chronic progressive external ophthalmoplegia (CPEO)	[159, 160]
12	Tetracycline	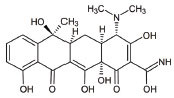	Antiapoptotic, antiinflammation, antioxidation and improves fitness	Mitochondrial dysfunction and Leigh syndrome	[160, 161]
13	Doxycycline	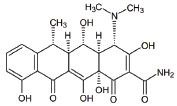	Prevents neuronal death and the accumulation of neuroimmune and inflammatory proteins	Mitochondrial dysfunction	[161]
14	Pioglitazone	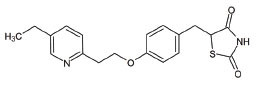	Peroxisome proliferator-activated receptor gamma (PPARγ) agonist	Pioglitazone improves lipopolysaccharide-induced behavioural loss, inflammation, white matter injury and mitochondrial dysfunction, inhibits diabetes-induced atrial mitochondrial oxidative stress and improves mitochondrial biogenesis, dynamics	[162, 163]
15	Sonlicromanol	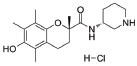	Improves neuronal network dysfunction	Mitochondrial encephalomyopathy, lactic acidosis, and stroke-like episodes (MELAS)	[164]
16	Oleanolic acid	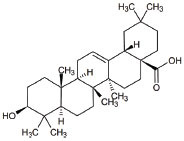	Rescued mitochondrial ultra-structure anomalies and mitochondrial biogenesis	Cardiac aging	[165]

**Table 3:** Mitochondria targeted biosimilar or bioengineered medicines other than ALS indications.

**S. No.**	**Interventions**	**Mechanism**	**Disease Condition**	**References**
1	Mitochondrial Replacement Therapy (MRT)	Restraint of mutated mitochondrial DNA transfer through mitochondrial donation procedures like maternal spindle transfer and pronuclear transfer in the oocyte or fertilized embryo is replaced with healthy copies of the mitochondrial genome	Mitochondrial dysfunction	[119, 167]
2	dNTPs therapy	Interruption of deoxyribonucleotide metabolism, which contributes to restricted availability of various deoxyribonucleoside triphosphates (dNTPs)	Mitochondrial DNA depletion syndrome (MDS) and Mitochondrial neuroastrointestinal encephalomyopathy (MNGIE)	[119, 168, 169]
3	Stem cell replacement/ transplantation therapies	Allogeneic Hematopoietic Stem Cell Transplant (HSCT)	Mitochondrial neurogastrointestinal encephalomyopathy (MNGIE)	[170, 171]
4	Gene therapy	Gene replacement therapy: Leber’s hereditary optic neuropathy (LHON), caused by mutations in the mitochondrially-encoded MT-ND4 gene	Leber’s hereditary optic neuropathy (LHON) is due to missense point mutations affecting mitochondrial DNA (mtDNA), usually found in the homoplasmic state, leading to mitochondrial dysfunction.	[172, 173]
5	Correction of DNA Heteroplasmy	Manipulating or selective elimination of mitochondrial DNA heteroplasmy by a mitochondrially targeted restriction endonuclease	Neuropathy, ataxia & retinitis pigmentosa (NARP), MILS syndromes	[174-176]
6	tRNAs synthase correction	Human mitochondrial leucyl-tRNA synthetase corrects mitochondrial dysfunctions due to the tRNALeu(UUR) A3243G mutation.	Mitochondrial encephalomyopathy, lactic acidosis, and stroke-like symptoms and diabetes	[177]
7	Elamipretide (MTP-131), a novel mitochondria-targeting tetrapeptide (D-Arg-dimethylTyr-Lys-Phe-NH2)	Stabilizes cardiolipin and enhances ATP synthesis in multiple organs, including the heart, kidney, neurons, and skeletal muscle	Impaired mitochondrial energy metabolism	[178]

## LIST OF ABBREVIATIONS

ADOA: Autosomal Dominant Optic Atrophy
ALS: Amyotrophic Lateral Sclerosis
ER: Endoplasmic Reticulum
FUS: Fused in Sarcoma
Hsp: Heat Shock Protein
KSS: Kearns-Sayre Syndrome
MAM: Mitochondria-associated Membranes
MCS: Membrane Contact Sites
MN: Motor Neuron
MQC: Mitochondrial Quality Control
NIBS: Non-invasive Brain Stimulation
PEO: Progressive External Ophthalmoplegia
TEM: Transmission Electron Microscope
UBA: Ubiquitin-associated
VCP: Valosin Containing Protein
